# Investigation of nitro–nitrito photoisomerization: crystal structure of *trans*-chlorido­nitro­(1,4,8,11-tetra­aza­cyclo­tetra­decane-κ^4^ *N*,*N*′,*N*′′,*N*′′′)cobalt(III) chloride

**DOI:** 10.1107/S205698901801678X

**Published:** 2018-11-30

**Authors:** Shigeru Ohba, Masanobu Tsuchimoto, Naoki Yamada

**Affiliations:** a Research and Education Center for Natural Sciences, Keio University, 4-1-1 Hiyoshi, Kohoku-ku, Yokohama 223-8521, Japan; b Department of Chemistry, Chiba Institute of Technology, Shibazono 2-1-1, Narashino, Chiba 275-0023, Japan; c Department of Chemistry, Faculty of Science and Technology, Keio University, Hiyoshi 3-14-1, Kohoku-ku, Yokohama 223-8522, Japan

**Keywords:** crystal structure, nitro-nitrito photo-isomerization, reaction cavity

## Abstract

The reaction cavity of the nitro group in the crystal of the title compound, [CoCl(NO_2_)(C_10_H_24_N_4_)]Cl, (I), was investigated to confirm that it offers sufficient free space for linkage isomerization to occur in accordance with the observed photochemical reactivity. The complex cation has crystallographic 2/*m* symmetry and the nitro and chloro ligands at the *trans* positions are statistically disordered. The complete cyclam ligand is generated by symmetry from a quarter of the mol­ecule. In the crystal of (I), the complex cations and Cl^−^ ions are linked into a three-dimensional network by N—H⋯Cl(counter-ion) hydrogen bonds.

## Abstract 2

The crystal structure of the title compound has been studied to show that the macrocyclic cyclam ligand is very suitable as the co-ligand for the nitro–nitrito photo-isomerization of the Co^III^ complex.

## Chemical context   

The photochemical reactions of metal complexes in the solid state attract much attention from crystallographers and chemists (Coppens *et al.*, 2002 ▸; Vittal & Quah, 2017 ▸). The present authors have investigated photochemical linkage isomerization of a series of the nitro­cobalt(III) complexes, *trans*-[Co(en)_2_(NO_2_)(NCS)]Cl·H_2_O and other salts, (Ohba, Tsuchimoto & Kurachi, 2018 ▸), *trans*-[Co(acac)_2_(NO_2_)(pyridine derivative)] (Ohba, Tsuchimoto & Miyazaki, 2018 ▸), and *trans*-[Co(salen)(NO_2_)(pyridine derivative)] (Ohba, Tsuchimoto & Yamada, 2018 ▸). In the present study, we describe our investigations of another type of nitro­cobalt complex, *trans*-[Co(cyclam)(NO_2_)Cl]Cl, (I), where cyclam stands for 1,4,8,11-tetra­aza­cyclo­tetra­decane. It is known that the stability of the nitrito–Co^III^ complexes greatly depends on the electronic effects of the co-existing ligands, and cyclam is expected to bring a small rate constant of the nitrito-to-nitro thermal reaction (Miyoshi *et al.*, 1983 ▸). The crystal structure of *trans*-[Co(cyclam)(NO_2_)_2_]ClO_4_, (II), has already been reported by Ohba *et al.* (2001 ▸). For (II) and the related PF_6_ salt, thermal conversion steps from the dinitrito to di­nitro form were investigated by differential scanning calorimetry and DFT calculations (Eslami *et al.*, 2014 ▸).

When a KBr disk of (I) was irradiated for 30 min with a Xe lamp, the IR spectrum showed an apparent change involving an increase in intensity of the absorption peak of *ca* 1000 cm^−1^ (see the Figure in the supporting information), which corresponds to the symmetric N—O stretching mode of the nitrito form (Eslami *et al.*, 2014 ▸). The IR spectrum of the irradiated complex was almost unchanged on standing at room temperature for 2 h, indicating the long life-time of the nitrito form as in (II), and reverted to that before irradiation by heating at 55°C for 45 min. The crystal structure of (I) was determined to establish the dimensions of the reaction cavity and steric circumstance of the nitro group, and to compare them with those in (II).
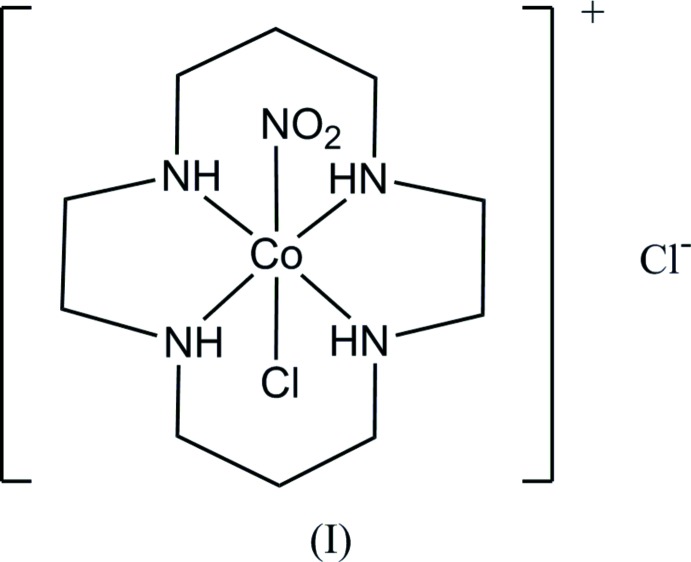
 
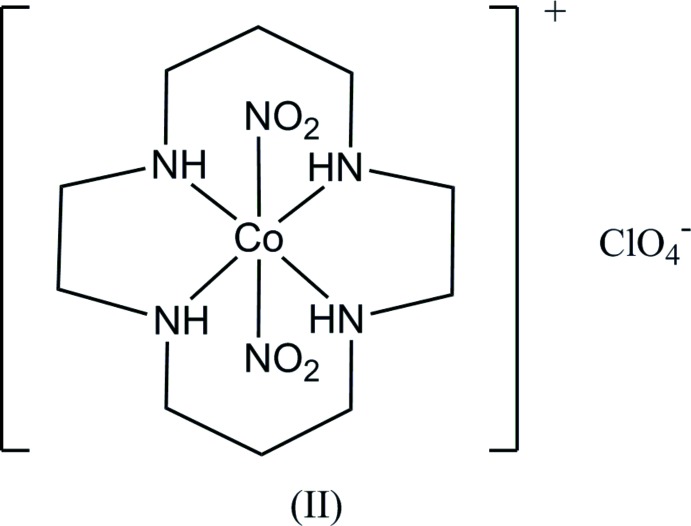


## Structural commentary   

**Figure 1:**
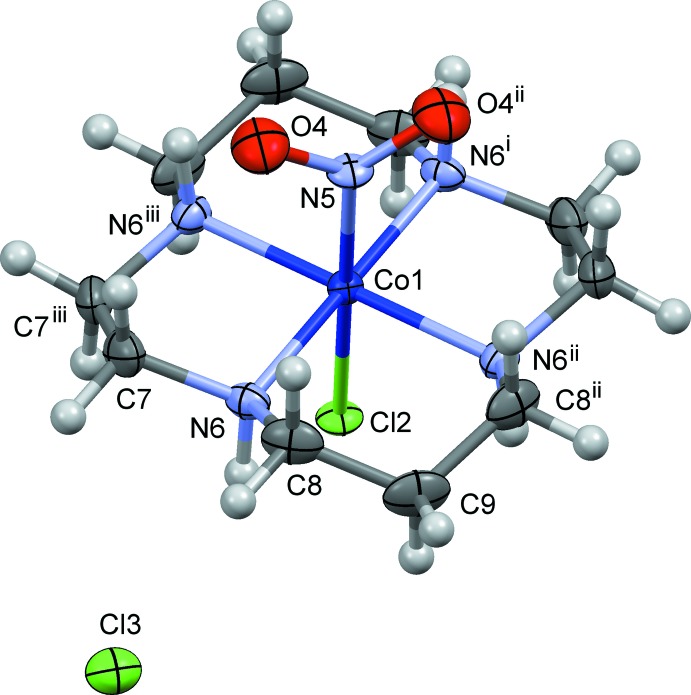
The mol­ecular structure of (I), showing displacement ellipsoids at the 30% probability level. A crystallographic twofold axis runs through atom Co1 and the midpoint of the C7—C7^iii^ bond. Only one of two possible orientations of the nitro and chloride ions is shown for clarity. Symmetry codes: (i) −*x* + 1, −*y*, −*z* + 1; (ii) *x*, *y*, −*z* + 1; (iii) −*x* + 1, −*y*, *z*.

The mol­ecular structure of (I) is shown in Fig. 1 ▸. The coordination geometry around the Co atom is a distorted octa­hedron with the N5 (nitro) and Cl2 atoms at the *trans* positions. The macrocyclic ligand cyclam adopts the *trans*-III conformation of Tobe’s classification (Bosnich *et al.*, 1965 ▸). The metal atom lies at site symmetry 2/*m*, and the atoms N5, Cl2 and C9 (the central C atom in the six-membered chelate ring) also lie on the mirror plane. There is a twofold axis running through the Co1 atom and midpoint of the C7—C7^iii^ bond in the five-membered chelate ring of cyclam, indicating that the positions of the Cl2 and nitro N5 atoms are exchanged. Similar orientational disorder of the chlorido­nitro­cobalt complexes is observed for *trans*-[Co(en)_2_Cl(NO_2_)]ClO_4_ (Ohba & Eishima, 2000*a* ▸) and the NO_3_ salt (Ohba & Eishima, 2000*b* ▸).

The Co—N(nitro) bond length is 1.9601 (10) Å, which is the result of restraint in the refinement of disorder, the length being similar to those in (II), 1.962 (5) and 1.968 (5) Å (Ohba, *et al.*, 2001 ▸). On the other hand, the Co—Cl bond distance is 2.2513 (12) Å, which is similar to that observed in *trans*-[Co(cyclam)Cl_2_]Cl, 2.2533 (4) Å (Ivaniková *et al.*, 2006 ▸). There are intra­molecular C—H⋯O/Cl hydrogen bonds (Table 1 ▸).

## Supra­molecular features   

**Figure 2:**
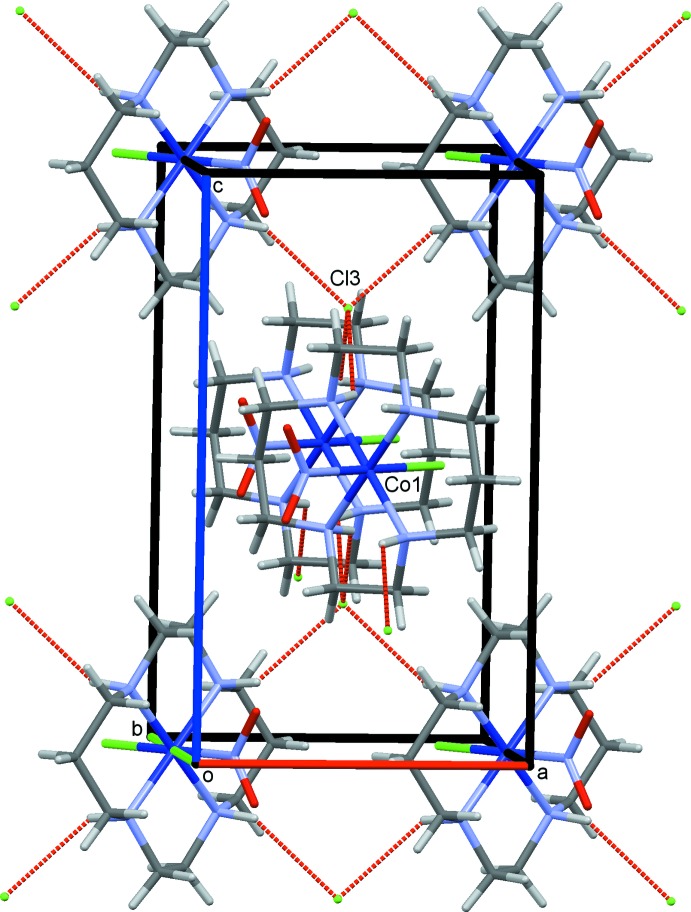
The crystal structure of (I), projected along *b*. The N—H⋯Cl hydrogen bonds are shown as red dashed lines. Only one of two possible orientations of the complex cation is shown for clarity.

**Figure 3:**
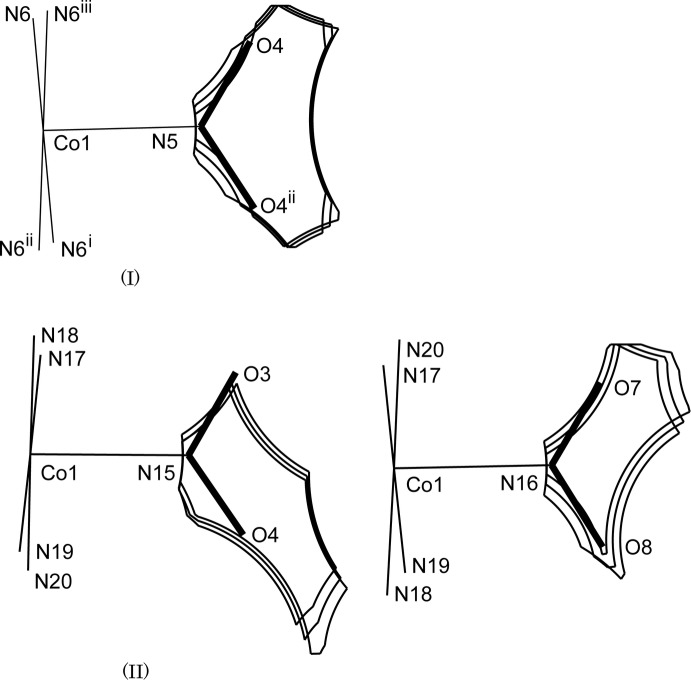
Comparison of the slices of the cavity around the nitro group within 0.1 Å from the plane in (I) and (II), where the minor O atoms (occupancy 35%) of each nitro ligand are omitted for clarity in (II). Symmetry codes for (I): (i) −*x* + 1, −*y*, −*z* + 1; (ii) *x*, *y*, −*z* + 1; (iii) −*x* + 1, −*y*, *z*.

**Figure 4:**
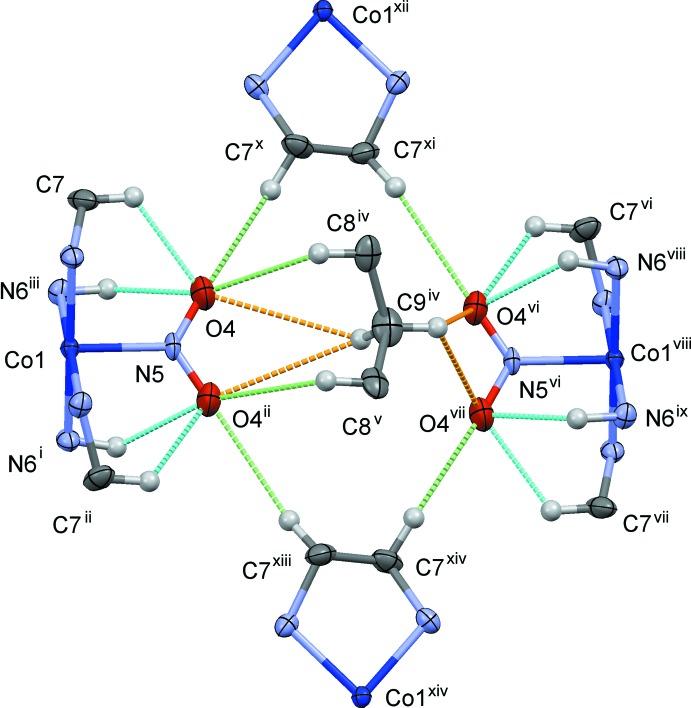
The steric circumstances of the nitro group in (I). Only parts of the complex are shown for clarity. The nitro group may be replaced with the Cl ligand due to the orientational disorder. There is a crystallographic twofold axis running vertical through the Co1^xii^ and Co1^xv^ atoms, another C8/C9 equivalent moiety which lies below the planes of the nitro groups being omitted for clarity. The N/C—H⋯O hydrogen bonds are shown as blue dashed lines. The other O⋯H contacts shorter than 2.9 Å (O4⋯H7*B^x^* = 2.78 Å and O4⋯H8*B* ^iv^ = 2.85 Å) are indicated as green dashed lines, and those of rather long distances (O4⋯H9*B* ^iv^ = 3.11 Å and O4^vi^⋯H9*A* ^iv^ = 3.09 Å) are indicated as orange dashed lines. Symmetry codes: (i) −*x* + 1, −*y*, −*z* + 1; (ii) *x*, *y*, −*z* + 1; (iii) −*x* + 1, −*y*, *z*; (iv) −*x*, −*y*, *z*; (v) −*x*, −*y*, −*z* + 1; (vi) −*x*, −*y* − 1, *z*; (vii) −*x*, −*y* − 1, −*z* + 1; (viii) *x* − 1, *y* − 1, *z*; (ix) *x* − 1, *y* − 1, −*z* + 1; (x) *y*, −*x*, −*z* + 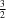; (xi) −*y*, *x* − 1, −*z* + 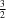; (xii) −*y*, *x* − 1, *z* + 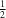; (xiii) *y*, −*x*, *z* − 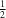; (xiv) −*y*, *x* − 1, *z* − 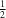.

**Figure 5:**
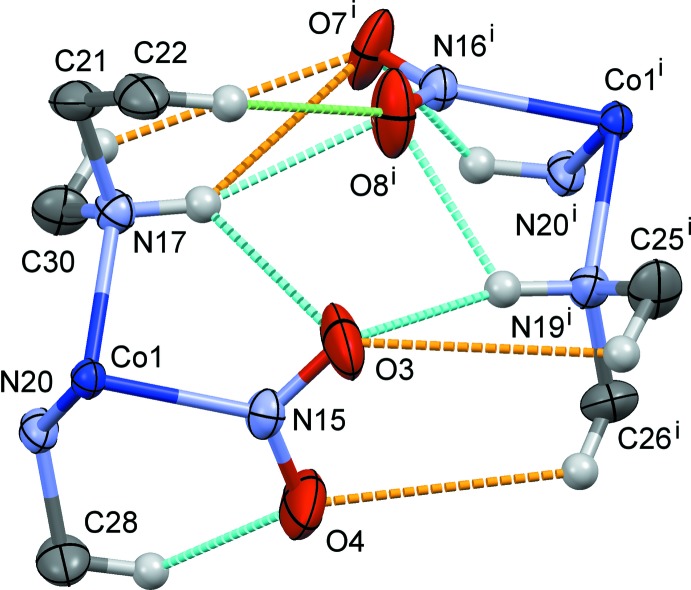
The steric circumstance of the nitro groups in (II). The minor O atoms (occupancy 35%) of each nitro ligand are omitted, and only parts of the complex are shown for clarity. The N/C—H⋯O hydrogen bonds are shown as blue dashed lines (H⋯O distances 2.13–2.29 Å). A green dashed line indicates the O8^i^⋯H22*B* contact of 2.63 Å. Other O⋯H contacts of rather long distances (3.03–3.09 Å) are indicated as orange dashed lines. Symmetry code: (i) *x*, *y*, *z* + 1.

The crystal structure of (I) is shown in Fig. 2 ▸. The complex cations and chloride ions are connected by N—H⋯Cl(counter-ion) hydrogen bonds, forming a three-dimensional network. In (II), there are two independent nitro ligands at the *trans* positions, and the O atoms of each nitro group show two possible positions (occupation factors 65 and 35%; Ohba, *et al.*, 2001 ▸). In the following discussion, the minor O(nitro) atoms will be neglected in (II). Slices of the reaction cavities around the NO_2_^−^ group near its plane in (I) and (II) are compared in Fig. 3 ▸, where the radii of neighboring atoms are assumed to be 1.0 Å greater than the corresponding van der Waals radii (Bondi, 1964 ▸), except for Co, its radius being set to 1.90 Å. The shape of the cavity in the nitro plane is mainly defined by the N/C—H⋯O(nitro) contacts which are shown in Figs. 4 ▸ and 5 ▸. Since the radius of the neighboring H atoms is assumed to be 2.20 Å, the cavity around the nitro O atoms is narrow in the intra- and inter­molecular hydrogen-bond directions. In (I), the cavity has sufficient free space to both side of the nitro O atoms for rotation to become the nitrito form, as suggested by the observed photoreactivity. In (II), the cavities of the nitro groups have space at one of the O atoms for conversion to the mono- and di-nitrito forms. The bifurcated N—H⋯O,O hydrogen bonds form an 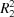
(4) ring (Fig. 5 ▸), which is also observed in the salts of *trans*-[Co(en)_2_(NO_2_)(NCS)]^+^ complexes (Ohba, Tsuchimoto & Kurachi, 2018 ▸)

## Database survey   

There is no entry for a (cyclam)nitro­cobalt(III) complex in the Cambridge Structural Database (CSD Version 5.39; Groom *et al.*, 2016 ▸), except for *trans*-[Co(cyclam)(NO_2_)_2_]ClO_4_ (Ohba *et al.*, 2001 ▸). The nitrito coordination was reported for certain Co^III^ complexes with cyclam derivatives, for example *trans*-[Co(Me_8_[14]ane)(ONO)_2_]ClO_4_ (Horn *et al.*, 2001 ▸), where Me_8_[14]ane stands for 3,10-*C*-*meso*-3,5,7,7,10,12,14,14-octa­methyl-1,4,8,11-tetra­aza­cyclo­tetra­decane, and *trans*-[Co(*L*)(NO_2_)(ONO)]ClO_4_ and *cis*-[Co(*L*)(NO_2_)(ONO)]ClO_4_ (Boyd *et al.*, 2007 ▸), where *L* stands for 1-(anthracen-9-ylmeth­yl)-1,4,8,11-tetra­aza­cyclo­tetra­decane.

The structures of *trans*-di­chloro complexes have been published for several salts, *i.e. trans*-[Co(cyclam)Cl_2_](Cl^−^)_1.47_(H_3_O^+^)_0.47_(H_2_O)_3.53_ (Sosa-Torres *et al.*, 1997 ▸), *trans*-[Co(cyclam)Cl_2_]Cl (Ivaniková *et al.*, 2006 ▸), *trans*-[Co(cyclam)Cl_2_]PF_6_ and *trans*-[Co(cyclam)Cl_2_]Tf_2_N, where Tf_2_N^−^ is bis­(tri­fluoro­methane­sulfon­yl)amide anion (Oba & Mochida, 2015 ▸), the conformation of cyclam in these crystals being *trans*-III according to Tobe’s classification (Bosnich *et al.*, 1965 ▸). The (cyclam)chloro­cobalt(III) alkynyl complexes such as *trans*-[Co(cyclam)Cl(1-ethynyl­naphthalene)]CF_3_SO_3_·OEt_2_ (Judkins *et al.*, 2018 ▸) have been studied for their structural and spectroscopic properties.

## Synthesis and crystallization   

*trans*-[Co(cyclam)Cl_2_]Cl was prepared by a literature method (Nakahara & Shibata, 1977 ▸) from cobalt(II) chloride hexa­hydrate and cyclam, and converted to *trans*-[Co(cyclam)Cl(NH_3_)]Cl_2_·H_2_O according to the method of Lee & Poon (1973 ▸). Then, *trans*-[Co(cyclam)Cl(NH_3_)]Cl_2_·H_2_O (1.0 mmol) was dissolved in 11 ml of 1% NH_3_ aqueous solution and neutralized with diluted HCl. To the solution sodium nitrite (8.0 mmol) and 1 ml of 1 *M* HCl were added, and the reaction mixture was stirred for 3 h at room temperature, and concentrated to precipitate the title compound, (I). Orange-red plate-like crystals of (I) were grown from a dimethyl sulfoxide solution by diffusion of diethyl ether vapour.

## Refinement   

Crystal data, data collection and structure refinement details are summarized in Table 2 ▸. The electron densities of the nitro N5 and Cl2 atoms overlap with each other because of the orientational disorder of the complex cation. An EADP command was used for atoms N5 and Cl2, and the Co1—N5 bond distance was restrained to be 1.960 Å (s.u. = 0.001 Å) to obtain a reasonable geometry for the nitro group. The H atoms bound to C and N were positioned geometrically. They were refined as riding, with C—H/N—H = 0.97–0.98 Å, and *U*_iso_(H) = 1.2*U*_eq_(C/N). One reflection showing poor agreement was omitted from the final refinement.

## Supplementary Material

Crystal structure: contains datablock(s) I, general. DOI: 10.1107/S205698901801678X/hb7787sup1.cif

Structure factors: contains datablock(s) I. DOI: 10.1107/S205698901801678X/hb7787Isup2.hkl

Supporting information file. DOI: 10.1107/S205698901801678X/hb7787Isup4.cdx

The IR spectra of chloro (I) and dinitro (II) complexes before and after photoirradiation for 30 min with a 150 W Xe lamp to the KBr disk, and after further standing for 2h.. DOI: 10.1107/S205698901801678X/hb7787sup3.tif

CCDC reference: 1881114

Additional supporting information:  crystallographic information; 3D view; checkCIF report

## Figures and Tables

**Table 1:** Hydrogen-bond geometry (Å, °)

*D*—H⋯*A*	*D*—H	H⋯*A*	*D*⋯*A*	*D*—H⋯*A*
N6—H6⋯Cl3	0.98	2.64	3.3671 (16)	131
C7—H7*A*⋯O4	0.97	2.28	2.929 (5)	124
C8—H8*B*⋯Cl2^i^	0.97	2.80	3.302 (4)	113

Symmetry code: (i) 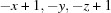.

**Table 2:** Experimental details

Crystal data	
Chemical formula	[CoCl(NO_2_)(C_10_H_24_N_4_)]Cl
*M*_r_	376.17
Crystal system, space group	Tetragonal, *P*4_2_/*m*
Temperature (K)	301
*a*, *c* (Å)	7.6052 (3), 13.3873 (7)
*V* (Å^3^)	774.31 (7)
*Z*	2
Radiation type	Mo *K*α
μ (mm^−1^)	1.46
Crystal size (mm)	0.25 × 0.25 × 0.10

Data collection	
Diffractometer	Bruker D8 VENTURE
Absorption correction	Integration (*SADABS*; Bruker, 2016 ▸)
*T*_min_, *T*_max_	0.704, 0.876
No. of measured, independent and observed [*I* > 2σ(*I*)] reflections	7967, 953, 922
*R*_int_	0.028
(sin θ/λ)_max_ (Å^−1^)	0.659

Refinement	
*R*[*F* ^2^ > 2σ(*F* ^2^)], *wR*(*F* ^2^), *S*	0.033, 0.079, 1.19
No. of reflections	953
No. of parameters	58
No. of restraints	1
H-atom treatment	H-atom parameters constrained
Δρ_max_, Δρ_min_ (e Å^−3^)	0.44, −0.26

Computer programs: *APEX3* and *SAINT* (Bruker, 2016 ▸), *SHELXT* (Sheldrick, 2015*a* ▸), *SHELXL2014* (Sheldrick, 2015*b* ▸), *Mercury* (Macrae *et al.*, 2008 ▸), *CAVITY* (Ohashi *et al.*, 1981 ▸) and *publCIF* (Westrip, 2010 ▸).

## supplementary crystallographic information

### Crystal data

**Table:**

[CoCl(NO_2_)(C_10_H_24_N_4_)]Cl	*D*_x_ = 1.613 Mg m^−^^3^
*M**_r_* = 376.17	Mo *K*α radiation, λ = 0.71073 Å
Tetragonal, *P*4_2_/*m*	Cell parameters from 6699 reflections
*a* = 7.6052 (3) Å	θ = 2.7–27.9°
*c* = 13.3873 (7) Å	µ = 1.46 mm^−^^1^
*V* = 774.31 (7) Å^3^	*T* = 301 K
*Z* = 2	Plate, orange
*F*(000) = 392	0.25 × 0.25 × 0.10 mm

### Data collection

**Table:**

Bruker D8 VENTURE diffractometer	922 reflections with *I* > 2σ(*I*)
φ and ω scans	*R*_int_ = 0.028
Absorption correction: integration (SADABS; Bruker, 2016)	θ_max_ = 27.9°, θ_min_ = 2.7°
*T*_min_ = 0.704, *T*_max_ = 0.876	*h* = −9→10
7967 measured reflections	*k* = −10→9
953 independent reflections	*l* = −15→17

### Refinement

**Table:**

Refinement on *F*^2^	Hydrogen site location: inferred from neighbouring sites
Least-squares matrix: full	H-atom parameters constrained
*R*[*F*^2^ > 2σ(*F*^2^)] = 0.033	*w* = 1/[σ^2^(*F*_o_^2^) + (0.0224*P*)^2^ + 0.627*P*] where *P* = (*F*_o_^2^ + 2*F*_c_^2^)/3
*wR*(*F*^2^) = 0.079	(Δ/σ)_max_ < 0.001
*S* = 1.19	Δρ_max_ = 0.44 e Å^−^^3^
953 reflections	Δρ_min_ = −0.26 e Å^−^^3^
58 parameters	Extinction correction: SHELXL2014 (Sheldrick, 2015b), Fc^*^=kFc[1+0.001xFc^2^λ^3^/sin(2θ)]^-1/4^
1 restraint	Extinction coefficient: 0.056 (7)
Primary atom site location: structure-invariant direct methods	

### Special details

**Table:**

Geometry. All esds (except the esd in the dihedral angle between two l.s. planes) are estimated using the full covariance matrix. The cell esds are taken into account individually in the estimation of esds in distances, angles and torsion angles; correlations between esds in cell parameters are only used when they are defined by crystal symmetry. An approximate (isotropic) treatment of cell esds is used for estimating esds involving l.s. planes.

### Fractional atomic coordinates and isotropic or equivalent isotropic displacement parameters (Å^2^)

**Table:**

	*x*	*y*	*z*	*U*_iso_*/*U*_eq_	Occ. (<1)
Co1	0.5000	0.0000	0.5000	0.02498 (19)	
Cl2	0.7410 (3)	0.1720 (4)	0.5000	0.0336 (3)	0.5
Cl3	0.5000	0.5000	0.7500	0.0529 (3)	
O4	0.2278 (5)	−0.2075 (6)	0.5775 (3)	0.0664 (11)	0.5
N5	0.2929 (11)	−0.1534 (15)	0.5000	0.0336 (3)	0.5
N6	0.4033 (2)	0.1478 (2)	0.60785 (13)	0.0352 (4)	
H6	0.4853	0.2464	0.6151	0.042*	
C7	0.4146 (4)	0.0476 (3)	0.70245 (16)	0.0519 (6)	
H7A	0.3180	−0.0353	0.7071	0.062*	
H7B	0.4085	0.1269	0.7591	0.062*	
C8	0.2278 (3)	0.2279 (3)	0.5944 (2)	0.0535 (6)	
H8A	0.2023	0.3034	0.6510	0.064*	
H8B	0.1396	0.1358	0.5927	0.064*	
C9	0.2167 (5)	0.3338 (5)	0.5000	0.0618 (11)	
H9A	0.3113	0.4194	0.5000	0.074*	
H9B	0.1066	0.3981	0.5000	0.074*	

### Atomic displacement parameters (Å^2^)

**Table:**

	*U*^11^	*U*^22^	*U*^33^	*U*^12^	*U*^13^	*U*^23^
Co1	0.0221 (3)	0.0253 (3)	0.0275 (3)	0.00039 (16)	0.000	0.000
Cl2	0.0237 (10)	0.0297 (8)	0.0476 (6)	−0.0111 (5)	0.000	0.000
Cl3	0.0454 (4)	0.0454 (4)	0.0678 (8)	0.000	0.000	0.000
O4	0.054 (2)	0.075 (3)	0.071 (2)	−0.0275 (19)	0.0046 (19)	0.004 (2)
N5	0.0237 (10)	0.0297 (8)	0.0476 (6)	−0.0111 (5)	0.000	0.000
N6	0.0349 (8)	0.0325 (8)	0.0383 (9)	−0.0020 (6)	0.0069 (7)	−0.0065 (7)
C7	0.0729 (17)	0.0515 (14)	0.0313 (10)	−0.0072 (11)	0.0128 (10)	−0.0039 (9)
C8	0.0368 (11)	0.0490 (13)	0.0746 (17)	0.0073 (9)	0.0170 (11)	−0.0152 (12)
C9	0.0433 (19)	0.0421 (18)	0.100 (3)	0.0182 (15)	0.000	0.000

### Geometric parameters (Å, º)

**Table:**

Co1—N5^i^	1.9601 (10)	N6—C7	1.481 (3)
Co1—N5	1.9601 (10)	N6—H6	0.9800
Co1—N6	1.9720 (16)	C7—C7^iii^	1.486 (6)
Co1—N6^i^	1.9720 (16)	C7—H7A	0.9700
Co1—N6^ii^	1.9720 (16)	C7—H7B	0.9700
Co1—N6^iii^	1.9720 (16)	C8—C9	1.501 (4)
Co1—Cl2	2.2513 (12)	C8—H8A	0.9700
Co1—Cl2^i^	2.2513 (12)	C8—H8B	0.9700
O4—N5	1.221 (4)	C9—C8^ii^	1.501 (4)
N5—O4^ii^	1.221 (4)	C9—H9A	0.9700
N6—C8	1.478 (3)	C9—H9B	0.9700
			
N5^i^—Co1—N5	180.0	O4^ii^—N5—Co1	121.76 (19)
N5^i^—Co1—N6	87.7 (3)	O4—N5—Co1	121.76 (19)
N5—Co1—N6	92.3 (3)	C8—N6—C7	111.64 (19)
N5^i^—Co1—N6^i^	92.3 (3)	C8—N6—Co1	118.86 (15)
N5—Co1—N6^i^	87.7 (3)	C7—N6—Co1	108.13 (13)
N6—Co1—N6^i^	180.0	C8—N6—H6	105.8
N5^i^—Co1—N6^ii^	87.7 (3)	C7—N6—H6	105.8
N5—Co1—N6^ii^	92.3 (3)	Co1—N6—H6	105.8
N6—Co1—N6^ii^	94.14 (10)	N6—C7—C7^iii^	107.55 (16)
N6^i^—Co1—N6^ii^	85.86 (10)	N6—C7—H7A	110.2
N5^i^—Co1—N6^iii^	92.3 (3)	C7^iii^—C7—H7A	110.2
N5—Co1—N6^iii^	87.7 (3)	N6—C7—H7B	110.2
N6—Co1—N6^iii^	85.86 (10)	C7^iii^—C7—H7B	110.2
N6^i^—Co1—N6^iii^	94.14 (10)	H7A—C7—H7B	108.5
N6^ii^—Co1—N6^iii^	180.0	N6—C8—C9	112.0 (2)
N5—Co1—Cl2	179.0 (5)	N6—C8—H8A	109.2
N6—Co1—Cl2	88.42 (7)	C9—C8—H8A	109.2
N6^i^—Co1—Cl2	91.58 (7)	N6—C8—H8B	109.2
N6^ii^—Co1—Cl2	88.42 (7)	C9—C8—H8B	109.2
N6^iii^—Co1—Cl2	91.58 (7)	H8A—C8—H8B	107.9
N5^i^—Co1—Cl2^i^	179.0 (5)	C8—C9—C8^ii^	114.7 (3)
N6—Co1—Cl2^i^	91.58 (7)	C8—C9—H9A	108.6
N6^i^—Co1—Cl2^i^	88.42 (7)	C8^ii^—C9—H9A	108.6
N6^ii^—Co1—Cl2^i^	91.58 (7)	C8—C9—H9B	108.6
N6^iii^—Co1—Cl2^i^	88.42 (7)	C8^ii^—C9—H9B	108.6
O4^ii^—N5—O4	116.4 (4)	H9A—C9—H9B	107.6
			
C8—N6—C7—C7^iii^	172.3 (2)	Co1—N6—C8—C9	−54.6 (3)
Co1—N6—C7—C7^iii^	39.7 (3)	N6—C8—C9—C8^ii^	67.3 (4)
C7—N6—C8—C9	178.5 (2)		

Symmetry codes: (i) −*x*+1, −*y*, −*z*+1; (ii) *x*, *y*, −*z*+1; (iii) −*x*+1, −*y*, *z*.

### Hydrogen-bond geometry (Å, º)

**Table:**

*D*—H···*A*	*D*—H	H···*A*	*D*···*A*	*D*—H···*A*
N6—H6···Cl3	0.98	2.64	3.3671 (16)	131
C7—H7*A*···O4	0.97	2.28	2.929 (5)	124
C8—H8*B*···Cl2^i^	0.97	2.80	3.302 (4)	113

Symmetry code: (i) −*x*+1, −*y*, −*z*+1.
