# Supplementary figures and images for: Investigation of nitro–nitrito photoisomerization: crystal structure of trans-chlorido­nitro­(1,4,8,11-tetra­aza­cyclo­tetra­decane-κ4 N,N′,N′′,N′′′)cobalt(III) chloride
Source: Acta Crystallogr E Crystallogr Commun. 2018 Nov 30;74(Pt 12):1908–12. doi: 10.1107/S205698901801678X (PMC6281089; doi:10.1107/S205698901801678X)

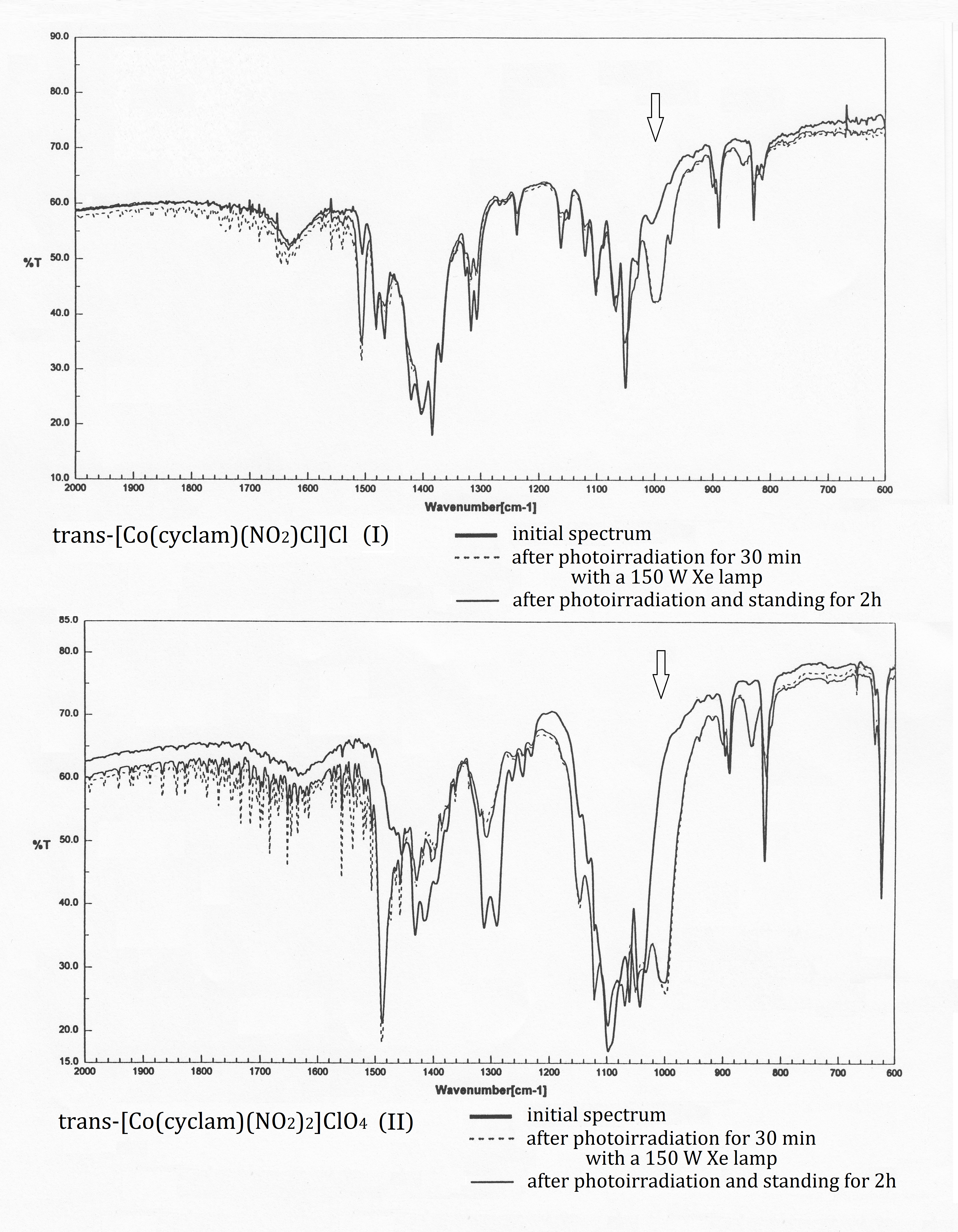

Supplement: Supplementary file 4 [file e-74-01908-sup3.tif]
